# LINC TOOL: USING QUALITY DATA, COACHING, AND AN IMPROVEMENT CYCLE FOR LEARNING AND IMPROVEMENT IN LONG-TERM CARE

**DOI:** 10.1093/geroni/igac059.1519

**Published:** 2022-12-20

**Authors:** Merel (E A ) Van Lierop, Judith Meijers, Erik van Rossum, Sandra Zwakhalen

**Affiliations:** Maastricht University, Maastricht, Limburg, Netherlands; Maastricht University, Maastricht, Limburg, Netherlands; Zuyd University of Applied Sciences, Maastricht, Limburg, Netherlands; Maastricht University, Maastricht, Limburg, Netherlands

## Abstract

Providing high quality of care can be challenging for nursing staff because of the continuous changes in healthcare. Nursing staff needs to learn continuously to remain employable and deal with these challenges. Therefore, availability of a continuous learning and improvement tool (LINC tool) using nursing quality data and an improvement cycle to maintain high quality of care in long-term care is important. The development of this tool took place in co-creation with researchers, experts, educational staff and nursing staff working in long-term care. Taskforces with researchers and experts on quality data and education were established to develop the main steps of the tool. Nursing staff was involved in the development and testing of the tool. The LINC tool consists of 1) Assessment of the current learning climate and preferred quality data for nursing staff to work with, 2) Discussing findings from step 1 with the nursing staff team and formulate learning topics, 3) Start improvement cycle (PDCA cycle) using one learning climate topic and one quality data topic as a case to improve quality of care and improve the learning climate. During this cycle, nurse coaches are available to help nursing staff use the LINC tool materials. The LINC tool can be used to improve long-term care and has proven to be feasible. However, the LINC tool is currently tested in more nursing staff teams to increase its usability and effectiveness. Further development of the LINC tool is therefore a constant cycle of developing, implementing, testing, and evaluating, all in cooperation with stakeholders.

